# Evolving models for peroxisome biogenesis^☆^

**DOI:** 10.1016/j.ceb.2014.02.002

**Published:** 2014-08

**Authors:** Ewald H Hettema, Ralf Erdmann, Ida van der Klei, Marten Veenhuis

**Affiliations:** 1 Department of Molecular Biology and Biotechnology, University of Sheffield, Western Bank, Sheffield S10 2TN, UK; 2 System Biochie, Ruhr Universitat Bochum, Universitatstr. 150, D-44780, Bochum, Germany; 3 Molecular Cell Biology, Groningen Biomolecular Sciences and Biotechnology institute, University of Groningen, 11 103, 9700CC, Groningen, The Netherlands

## Abstract

Significant progress has been made towards our understanding of the mechanism of peroxisome formation, in particular concerning sorting of peroxisomal membrane proteins, matrix protein import and organelle multiplication. Here we evaluate the progress made in recent years. We focus mainly on progress made in yeasts. We indicate the gaps in our knowledge and discuss conflicting models.

**Current Opinion in Cell Biology** 2014, **29**:25–30

This review comes from a themed issue on **Cell organelles**

Edited by **David K Banfield** and **Will Prinz**

For a complete overview see the Issue and the Editorial

Available online 28th March 2014

0955-0674/$ – see front matter, Crown Copyright © 2014 Published by Elsevier Ltd. All rights reserved.

**http://dx.doi.org/10.1016/j.ceb.2014.02.002**

## Introduction

Peroxisomes are eukaryotic organelles bound by a single membrane. Their abundance and functions vary between organisms, cell types and environmental conditions. In a seminal review, Lazarow and Fujiki [1] proposed that peroxisomal membrane and matrix proteins are synthesized on free polyribosomes and imported posttranslationally into preexisting peroxisomes. The endoplasmic reticulum was presumed to synthesise the membrane phospholipids of peroxisomes. Thus, they were considered autonomous organelles like mitochondria and chloroplasts. Lazarow and Fujiki stated that one of the implications of this model is that peroxisomes never form *de novo* [1].

Genetic screens in the late 80s and 90s identified many factors required for import of peroxisomal matrix proteins. The growth and division model was challenged by the discovery that mutants that appear to lack peroxisomal membranes can form peroxisomes *de novo* from the ER upon complementation. Since then the ER has been central to studies on peroxisome biogenesis. Below we assess the recent literature on peroxisomal matrix protein import and membrane formation.

## Posttranslational import of matrix proteins

Protein import into peroxisomes differs from protein import into most other organelles as (1) peroxisomes import folded and even oligomeric proteins and (2) peroxisomal import receptors cycle between a soluble, free form in the cytosol and a cargo-loaded form at the peroxisomal membrane, which is associated with the translocon, and at the end of the cycle is ubiquitinated and released from the membrane in an ATP-dependent process [2]. Peroxisomal matrix proteins contain specific peroxisomal targeting signals (PTS1 or PTS2) that are post-translationally recognized in the cytosol by the import receptors Pex5 and Pex7, respectively [3, 4, 5, 6, 7, 8••].

**Figure 1:**
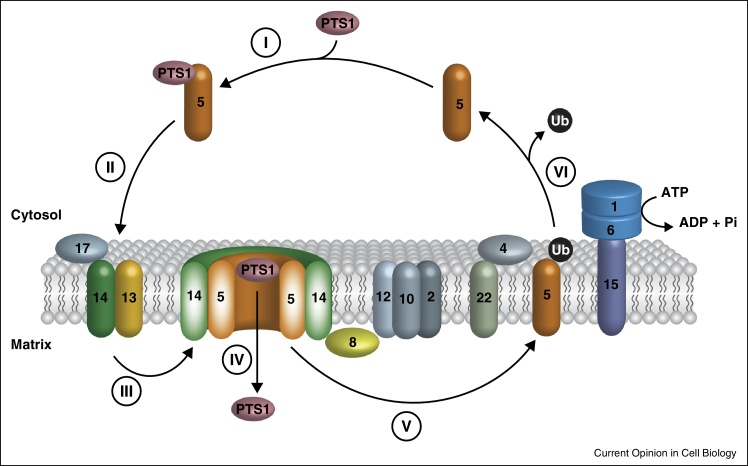
Model of peroxisomal matrix protein import. **(I)** Proteins harboring a peroxisomal targeting signal of type 1 (PTS1) are recognized and bound by the import receptor Pex5 in the cytosol. **(II)** The cargo-loaded receptor is directed to the peroxisomal membrane and binds to the docking complex (Pex13/Pex14/Pex17). **(III)** The import receptor assembles with Pex14 to form a transient pore and **(IV)** cargo proteins are transported into the peroxisomal matrix in an unknown manner. Cargo release might involve the function of Pex8 or Pex14. **(V)** The import receptor is monoubiquitinated at a conserved cysteine by the E2-enzyme complex Pex4/Pex22 in tandem with E3-ligases of the RING-complex (Pex2, Pex10, Pex12). **(VI)** The ubiquitinated receptor is released from the peroxisomal membrane in an ATP-dependent manner by the AAA-peroxins Pex1 and Pex6, which are anchored to the peroxisomal membrane *via* Pex15. As the last step of the cycle, the ubiquitin moiety is removed and the receptor enters a new round of import. The designation is based on the yeast nomenclature.

Receptor-cargo complexes are directed to a docking complex at the peroxisomal membrane (see Figure 1). The PTS1 receptor mediates cargo binding as well as association with the import machinery, whereas the PTS2 receptor binds cargo but requires auxiliary proteins such as PEX5L in mammals and plants, or proteins of the Pex18 family in fungi for membrane association and cargo translocation [2]. The crystal structure of Pex7 in complex with the Pex18 paralog Pex21 and a PTS2 peptide visualizes the cooperative binding mode of the PTS2 pre-import complex [8^••^].

The cargo-loaded receptor is thought to assemble with components of the docking complex to form the translocon, which allows translocation of folded proteins across the peroxisomal membrane into the matrix. The current opinion is based on the concept of a transient pore that assembles at the peroxisomal membrane and is disassembled after import, with its components being recycled for further rounds of protein import [9]. The major constituents of the dynamic pore for PTS1 import are the PTS1 receptor and the PMP Pex14: this constitutes the minimal functional unit for translocation of matrix proteins *in vivo* [10], and in electrophysiological studies it displays features of a regulated pore [11]. A major question is whether PTS1 and PTS2 proteins are imported *via* common or distinct import pores. Also currently debated is whether the cargo-loaded receptor remains associated with the pore (shuttle hypothesis) or whether it is released as a soluble receptor-cargo complex into the peroxisomal matrix (extended shuttle hypothesis). Once the cargo has reached the peroxisomal matrix, it is released from the receptor, which may require the intraperoxisomal peripheral membrane protein Pex8 [2, 12] or Pex14 [13]. It is unknown how folded proteins are translocated through the pore. Moreover, the exact composition of the pore as well as the driving force for cargo translocation remained elusive. During or after dissociation of the receptor-cargo complex, the PTS1 receptor is mono-ubiquitinated at a conserved cysteine, which serves as a signal for ATP-dependent dislocation of the receptor from the membrane to the cytosol [14]. It remains to be investigated why a cysteine and not a lysine is the evolutionarily conserved residue. Interestingly, in the PTS2-pathway, Pex7 but the auxiliary Pex18 family proteins are mono-ubiquitinated, again at a conserved cysteine [15, 16].

Mono-ubiquitination of receptors is performed by the peroxisomal ubiquitin-conjugating enzyme Pex4 [17]. Mono-ubiquitination is thought to prime the receptor for recognition by a complex of the AAA-type ATPases Pex1 and Pex6, which functions as dislocase to release the modified receptor from the membrane to the cytosol [18, 19]. AWP1 has been identified as a cofactor of mammalian Pex6 that binds mono-ubiquitinated Pex5 and is involved in the regulation of Pex5 export [20]. During or shortly after export, the PTS1 receptor is deubiquitinated and thus made available for another round of import [21, 22].

The mechanism of how folded proteins are translocated through the peroxisomal pore and also the driving force for this process remain elusive. The tagging of a substrate by monoubiquitylation or polyubiquitylation and its subsequent recognition and ATP-dependent removal from a membrane by ATPases of the AAA-family of proteins, resembles the mechanism of the membrane release of proteins for ER-associated degradation (ERAD) [23, 24]. As the ATP-dependent release of the ubiquitinated peroxisomal receptor from the membrane is the energy-requiring step of the matrix protein import cascade, the peroxisomal AAA peroxins might induce conformational changes of the receptor in an ATP-dependent manner that allows receptor release and cargo translocation. This link of receptor export and protein import is described by the export-driven import model [23].

## Different mechanisms of PMP sorting

Until recently, two classes of PMPs were distinguished based on their Pex19 dependence for targeting to peroxisomes [25]. Class 1 PMPs contain targeting information that is recognized by Pex19, which binds newly synthesized PMPs in the cytosol and acts as a chaperone/targeting receptor, thus (1) preventing them from aggregating and (2) delivering them to the peroxisomal membrane for docking on Pex3 [26, 27, 28]. Although posttranslational targeting of PMPs to peroxisomes *in vivo* and *in vitro* is well documented [25, 28, 29, 30, 31, 32••], the mechanism of insertion remains unclear. The central role of Pex3 and Pex19 in PMP biogenesis is evident from the phenotype of *pex3* and *pex19* mutants, in which peroxisomal membranes are undetectable and class I PMPs are unstable or mislocalised [33]. On the basis of these observations we proposed that these mutants lack any peroxisomal structures.

**Figure 2:**
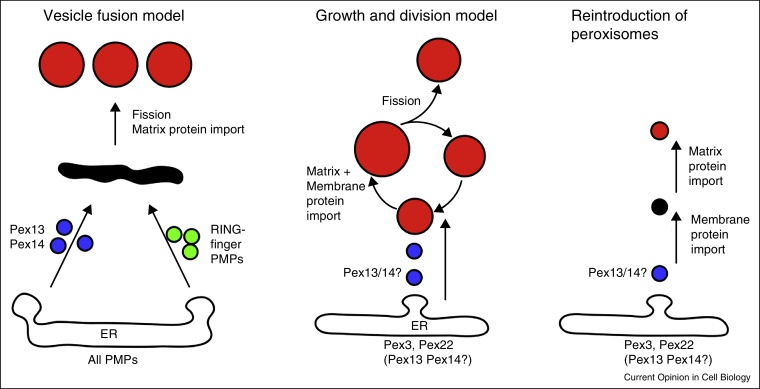
Schematic representation of models for peroxisome multiplication. The **vesicle fusion model** proposes that all PMPs enter the ER, where they segregate and exit the ER in distinct vesicles: vesicles containing the docking complex proteins Pex13,14 (blue) fuse with vesicles containing the RING-finger complex proteins Pex2,10,12 (green) forming a preperoxisomal membrane structure (black). As this membrane structure now contains a complete importomer, matrix protein import commences. Subsequent fission results in peroxisomes of final size and membrane protein composition (red). The **growth and division model** proposes that peroxisomes (red) are derived from existing peroxisomes by fission. A small subset of PMPs (Class 2) insert into the ER and exit it in a transport vesicle (blue) that fuses with existing peroxisomes, where it provides the docking site for Pex19-mediated import of Class 1 PMPs (Class 1). Since Pex3 can only be detected in peroxisomes in wild type cells it cannot be excluded that it inserts directly into peroxisomes. **Reintroduction of Peroxisomes.** In the absence of pre-existing peroxisomes, the ER-derived Pex3-containing vesicle matures slowly into a peroxisome, with Pex3 again providing a docking site for Pex19/Class I PMPs complexes; this vesicle is thus slowly converted into a membrane structure containing all PMPs (black), finally becoming import-competent for matrix proteins (red). These newly formed peroxisomes will further multiply by growth and division. Whether Pex13 and Pex14 traffic *via* the ER or insert directly into a membrane structure distinct from the ER is not established.

Class 2 PMPs, the best-studied example of which is Pex3, contain targeting signals that are not recognized by Pex19. Upon *de novo* formation of peroxisomes by induction of Pex3 expression in *S. cerevisiae pex3* cells, most data support a model whereby Pex3 enters the ER and forms preperoxisomes that develop into peroxisomes [34, 35, 36]. Its targeting and subsequent sorting signals have been described [37, 38], and the ER translocon has been implicated in this process [37]. *In vitro* budding reactions release vesicles containing Pex3, dependent on Pex19, cytosol and ATP [39, 40]. Budding occurs independently of the machinery required for ER exit of secretory proteins. These vesicles therefore might represent transport vesicles that fuse with pre-existing peroxisomes, thereby delivering Pex3 and lipids to peroxisomes (Figure 2).

As Pex3 is not observed in the ER of WT cells, one has to propose that its transit through the ER is fast. Thus far, Pex3 has been visualized in the ER only after overexpression, or by using mutant versions appended with large tags in cells blocked in peroxisome formation (*pex19* or *pex3* cells). It is therefore possible that yeast Pex3 bypasses the ER in wild type cells and is inserted directly into the peroxisomal membrane as has been reported for human Pex3 [41]. In line with this is the study by Knoops *et al.* [42]. They could detect no ER pool of Pex3 after its reintroduction into *pex3* cells and propose that *H. polymorpha* Pex3 bypasses the ER altogether (see also below). This is an issue that needs further investigation.

Recently, detailed microcopy studies challenged the view that *pex3* and *pex19* cells are devoid of peroxisomal structures with the finding that the *H. polymorpha pex3* and *pex19* cells contain pre-peroxisomes to which Pex13 and Pex14 localise [42]. This suggests that Pex13 and Pex14 represent a third class of PMPs that also sort independent of Pex3 and Pex19. Pex13-containing membrane structures have been found in *Pichia pastoris pex3* cells [43], and in *S. cerevisiae pex3* and *pex19* cells [44]. Furthermore, mammalian Pex13 inserts into membranes independent of Pex19 [45]. Also, Pex13 is required for Pex14 sorting [46], underlining that these PMPs behave differently from class I and II PMPs.

## Peroxisome multiplication and the contribution of the ER

In wild type yeast cells, peroxisomes receive newly synthesized membrane and matrix proteins and lipids (growth) and multiply by fission [38, 47, 48, 49••, 50]. Peroxisome fission is mediated by dynamin-related proteins and Pex11 (for recent review see [51]). Most peroxisomal membrane lipids are synthesized in the ER. They may be directly transferred from the ER to peroxisomes [59] or reach peroxisomes *via* vesicular transport. However, it has also been proposed that peroxisomal membranes derive from the ER *via* budding of vesicles containing PMPs. Tabak and coworkers recently reported that all newly synthesized *S. cerevisiae* PMPs insert into the ER, and that the Pex13/14 docking complex and the RING-finger complex exit the ER in distinct vesicles [44, 52]. These ‘half importomer’ complexes were proposed to be brought together by heterotypic vesicle fusion dependent on Pex1 and Pex6, forming a pre-peroxisomal membrane structure able to import matrix proteins. Subsequent fission would produce peroxisomes of final size and membrane composition (Figure 2). Besides being responsible for *de novo* formation of peroxisomes in peroxisome-deficient mutant cells, Tabak and coworkers claim that this mechanism also provides a ‘continuous stream’ of *de novo* formed peroxisomes in wild type cells [52, 53]. Although elegant, this model is irreconcilable with many of the independent studies described above. This model disregards for instance the well established role of Pex19-dependent posttranslational insertion of newly synthesized PMPs directly into peroxisomes [25, 26, 27, 28, 29, 30, 31, 32••], the mislocalisation of many *S. cerevisiae* PMPs to the cytosol in *pex3* and *pex19* cells [33], and that new peroxisomes are derived from preexisting peroxisomes [47, 48, 49••, 50]. Furthermore, Pex25 is required for *de novo* peroxisome formation, but most *pex25* cells contain peroxisomes, which suggests the *de novo* pathway is not essential for peroxisome maintenance [54••, 55]. Additionally, the studies by Knoops *et al.* [42] indicated that not the ER, but the Pex13/Pex14-containing structures present in *H. polymorpha pex3* cells are the target for reintroduced Pex3. Although the origin of the Pex13/Pex14 preperoxisomal structures is unclear, their formation is independent of Pex3.

The shape of the ER appears to affect peroxisome multiplication. Pex30 is a Class 2 PMP [56] that localises to both peroxisomes and an ER subdomain found in close association with peroxisomes [57, 58••]. It has been postulated that new peroxisomes can form from these ER subdomains. Interestingly, COPI components were found in complex with Pex30, although the functional significance of this is unclear. The ER reticulons Rtn1, Rtn2 and Ypo1 were identified as core components of Pex30 complexes. ER reticulons are important for maintaining the morphology of tubular ER by stabilizing the strongly curved membranes. Interestingly, in cells lacking the ER reticulons or Pex30, *de novo* formation of peroxisomes is accelerated [58^••^]. Whether these mutants form peroxisomes *de novo* all the time is not known.

## A multi-step model for peroxisome biogenesis

There is overwhelming evidence in favor of a growth and division model, whereby the insertion machinery for Class1 PMPs is central. As Pex3 is present at steady-state on peroxisomes in wild type cells, Pex19 bound to newly synthesized Class 1 PMPs may dock here to deliver them. Class 2 PMPs, including Pex3, traffic *via* the ER and vesicular transport to peroxisomes. This route may provide sufficient membrane constituents for continuing growth and division of peroxisomes, although a non-vesicular membrane transfer may contribute as well [59]. In cells forming peroxisomes *de novo*, Pex3 first inserts in the ER and sorts to preperoxisomes, where it facilitates Class 1 PMP insertion, allowing these membrane structures to import matrix proteins and mature into peroxisomes. Pex13 and Pex14 may follow the Pex3 route in cells synthesizing peroxisomes *de novo*, or they may bypass the ER and insert directly into preperoxisomes that can form independently of Pex3. Whether Pex13 and Pex14 follow this route in wild type cells is an important question and remains to be determined.

## Concluding remarks

The global mechanisms of peroxisome membrane biogenesis and matrix protein import are becoming clear, but many questions remain. To mention but a few: how are ER-inserted PMPs sorted away from the secretory pathway? How do PMPs leave the ER, and how are they delivered specifically to peroxisomes? Do Class1 PMPs insert spontaneously into peroxisomal membranes after targeting, or is a specialised machinery required? How is peroxisome size and number regulated? Which proteins make up the pores for matrix protein import, and how is matrix protein translocation mediated? New approaches will need to be developed to shed light on these questions.

## References and recommended reading

Papers of particular interest, published within the period of review, have been highlighted as:

• of special interest
•• of outstanding interest
